# Biotransformation of Animal Fat-By Products into ARA-Enriched Fermented Bioproducts by Solid-State Fermentation of *Mortierella alpina*

**DOI:** 10.3390/jof6040236

**Published:** 2020-10-21

**Authors:** Ondrej Slaný, Tatiana Klempová, Volha Shapaval, Boris Zimmermann, Achim Kohler, Milan Čertík

**Affiliations:** 1 Institute of Biotechnology, Faculty of Chemical and Food Technology, Slovak University of Technology, Radlinského 9, 812 37 Bratislava, Slovakia; ondrej.slany@stuba.sk (O.S.); milan.certik@stuba.sk (M.Č.); 2 Faculty of Science and Technology, Norwegian University of Life Sciences, Postbox 5003, 1432 Ås, Norway; volha.shapaval@nmbu.no (V.S.); boris.zimmermann@nmbu.no (B.Z.); achim.kohler@nmbu.no (A.K.)

**Keywords:** *Mortierella alpina*, solid-state fermentation, animal fat by-product, arachidonic acid, ATR-FTIR spectroscopy

## Abstract

Solid-state fermentation (SSF) is a powerful fermentation technology for valorizing rest materials and by-products of different origin. Oleaginous *Zygomycetes* fungi are often used in SSF as an effective cell factory able to valorize a wide range of hydrophilic and hydrophobic substrates and produce lipid-enriched bioproducts. In this study, for the first time, the strain *Mortierella alpina* was used in SSF for the bioconversion of animal fat by-products into high value fermented bioproducts enriched with arachidonic acid (ARA). Two cereals-based matrixes mixed with four different concentrations of animal fat by-product were evaluated for finding optimal conditions of a fat-based SSF. All obtained fermented bioproducts were found to be enriched with ARA. The highest substrate utilization (25.8%) was reached for cornmeal and it was almost double than for the respective wheat bran samples. Similarly, total fatty acid content in a fermented bioproduct prepared on cornmeal is almost four times higher in contrast to wheat bran-based bioproduct. Although in general the addition of an animal fat by-product caused a gradual cessation of ARA yield in the obtained fermented bioproduct, the content of ARA in fungal biomass was higher. Thus, *M. alpina* CCF2861 effectively transformed exogenous fatty acids from animal fat substrate to ARA. Maximum yield of 32.1 mg of ARA/g of bioproduct was reached when using cornmeal mixed with 5% (*w*/*w*) of an animal fat by-product as substrate. Furthermore, implementation of attenuated total reflectance Fourier transform infrared (ATR-FTIR) spectroscopy in characterization of obtained SSF bioproducts was successfully tested as an alternative tool for complex analysis, compared to traditional time-consuming methods.

## 1. Introduction

In Europe, approximately 16 million tonnes of animal fat (AF) by-products are processed annually by meat processors and renderers [1]. The total amount of AF by-products is expected to increase continuously, since consumers and health authorities in European countries focus on highly unsaturated fat diets [2]. In addition, due to the population growth, meat production in Europe is expected to grow. Even though some of these AF by-products comply with EU standards (EC No. 1069/2009) and can be served for human consumption, they are used almost exclusively as ingredients of animal feed. The biggest problem lies in the composition of AF by-products since they are rich in saturated and monounsaturated fatty acids. Long-term consumption of saturated fat elevates the risk of certain diseases such as diabetes or cardio-vascular problems. Thus, human food, as well as animal and aquaculture feeds, need to be supplied with the essential polyunsaturated ω-3 and ω-6 fatty acids (PUFAs), such as linoleic acid (LA; C18:2, ω-6), α-linolenic acid (ALA; C18:3, ω-3), arachidonic acid (ARA; C20:4, ω-6), eicosapentaenoic acid (EPA, C20:5, ω-3) and docosahexaenoic acid (DHA, C22:6, ω-3) [3]. Therefore, the development of sustainable processes for valorizing AF by-products into high-value bioproducts is urgently needed.

Fungal fermentation is an emerging technology for upgrading different types of by-products [4,5]. A certain group of filamentous fungi can accumulate up to 85% (*w*/*w*) of triacylglycerols (TAGs) containing high amount of essential PUFAs as a storage material of the cells [6]. Fungal biomass produced with these oleaginous fungi contains single-cell oils with a high share of essential ω-3 and ω-6 PUFAs, and is considered a high-value bioproduct with applications in food, feed and nutraceutical industry. Fungal biomass can be obtained by two fermentation processes: submerged or solid-state fermentation (SSF).

SSF is a fermentation technology using solid or semi-solid substrates in the absence of any water or using a low level of free-flowing water [7]. SSF has many advantages over more common submerged fermentation: (1) low energy requirements are accompanied by the high product yield; (2) downstream costs are significantly lower due to the high product applicability; (3) SSF conditions are more favorable for the microbial growth as they resemble the natural environment of microorganisms, resulting in a better performance of the fermentation process; (4) due to the high product/volume productivity of SSF, smaller fermentation volumes are possible [8]; (5) consumption of a lower amount of water and generation of low to almost zero amount of waste [9]. Solid substrates used in SSF frequently originate from agro-industrial waste materials and they serve as support materials for the optimal fungal growth, proliferation and single-cell oil production. Commonly used SSF substrates are cereal-based materials, such as barley flakes, millet grain, wheat bran, cornmeal or oat flakes [10,11]; fruit or vegetable-based materials, such as pulp, pomaces and peels (i.e., residues from plums, pineapple, carrot, papaya etc.) [12,13]; or they often come as residue from other types of industry, for example spent malt grain, groundnut fodder, forestry rests or wooden sawdust [7,14]. Several studies investigated the suitability of different oleaginous fungi, belonging to orders *Mucorales* and *Mortierellales*, for converting various waste materials and by-products based substrates by SSF into fermented BPs enriched with a wide range of essential PUFAs, such as γ-linolenic acid (GLA; C18:3, n-6), ARA or EPA [10,12,15,16,17,18]. Especially cereal-based solid matrixes are very suitable for these oleaginous fungi due to their chemical composition, such as the presence of easily accessible carbon, organic nitrogen and other macro- and micro-nutrients. Cereal-based matrixes provide a useful source for good fungal proliferation, hyphae penetration and stable lipid accumulation in fungal cells [10,15,16].

Oleaginous fungus *Mortierella alpina* is a well-known and thoroughly described species with a high capacity of lipid accumulation and good ability to produce industrially relevant essential PUFAs, especially ARA in both submerged fermentations and SSF [19,20,21]. It has also been reported that *M. alpina* is able to incorporate and transform exogenous fatty acids [22]. Thus, in order to improve the conversion of solid wastes into ARA-enriched bioproduct, supplementation with exogenous oils containing precursors of ARA is advised. Thus, the addition of vegetable oils, such as sunflower, rapeseed, corn, soybean or linseed oil containing individual fatty acid precursors of ARA led to a rapid increase of ARA yield in the final fermented bioproduct obtained by fungal SSF [10]. Such knowledge led to the hypothesis that the oleaginous fungus *M. alpina* should also be able to utilize and convert solid lipidic waste, such as AF by-products.

When developing a new SSF process and optimizing SSF processes, it is crucial to monitor key process parameters, such as humidity, airflow, oxygen transfer and controlling the quality of final fermented bioproduct. Quality parameters of the final fermented BP are fatty acid profile, total lipid content, amount of fungal biomass and total biochemical profile. For the determination of fatty acid profile and content, gas chromatography coupled with flame ionization detector (GC-FID) is the most commonly used technique [23], while the total amount of fungal biomass obtained by SSF is frequently analyzed by the glucosamine method [24]. Fourier transform infrared (FTIR) spectroscopy is a new emerging technique that has already been extensively used for characterizing biomass from different types of submerged cultivations [25,26,27,28]. FTIR spectroscopy is a biophysical technique allowing to measure the biomass in its native form. It is non-destructive and rapid and does not require extraction of single components. Therefore, the characterization of a bioproduct obtained in the process of SSF using FTIR spectroscopy could bring the new possibilities in complex monitoring of SSF process.

To the authors knowledge, this is the first study investigating the possibility to develop an efficient bioconversion of AF by-products into functional lipid-rich fermented bioproduct by SSF using *M. alpina*. Furthermore, the presented study introduces, describes and evaluates FTIR spectroscopy as an alternative method for accurate SSF monitoring.

## 2. Materials and Methods

### 2.1. Production Microorganism and Preparation of Spore Suspension

The oleaginous fungal strain *Mortierella alpina* CCF2861 used in this study was obtained from culture collection of fungi (CCF, Charles University, Prague, Czech Republic). The strain was kept on potato-dextrose agar media (PDA, Carl Roth, Germany) at 4 °C and regularly re-inoculated every 3 months.

The spore suspension for the inoculation of the fermentation substrate was prepared from a 14-day-old culture grown on PDA medium. Spores have been washed using an aqueous solution of 0.05% Tween^®®^ 40 and suspension was diluted to achieve a final concentration of 10^6^ spores/mL.

### 2.2. Conditions of Solid-State Fermentation

SSF cultivation was performed in the microporous high-density polyethylene bags (20 × 30 cm) containing 20 g of dry cereal-based matrix, to which various amounts of AF by-products (Norilia, Oslo, Norway) mixed with Tween^®®^ 40 (Sigma-Aldrich, Darmstadt, Germany) were added. SSF experiments were performed using two waste cereal-based matrixes: cornmeal (CM; Amylum Slovakia, Boleráz, Slovakia) and wheat brans (WB; Mill Pohronský Ruskov, Pohronský Ruskov, Slovakia). The composition of AF used is summarized in Table 1.

The cereal materials were soaked for 2 h in 20 mL (ratio solid to liquid 1:1) of either distilled water, Tween 40^®®^ solution or AF emulsion, as per Table 2. After 2 h, all SSF substrates in bags were autoclaved (105 °C, 45 min). After cooling down, the substrates were inoculated with 4 mL of spore suspension of *M. alpina*. The fermentation ran for 10 days at 20 °C. In order to maintain the homogenous growth of fungi, substrates were mixed thoroughly after inoculation. During the fermentation process, the whole content of the fermentation bags was gently mixed once a day. All experiments were performed in three independent biological replicates.

### 2.3. Preparation of AF Emulsion

Due to the fact that cereal-based matrixes have a hydrophilic nature while AF is hydrophobic, there is a need to perform the pretreatment of the fat materials before mixing it with cereal matrixes for obtaining homogenous substrates for SSF. The pretreatment of AF by-product was performed by preparing homogenous fat-water-Tween^®®^ 40 emulsions, where the emulsifier Tween^®®^ 40 formed a film around the dispersed lipid droplets and thereby reduced interfacial tension [29].

AF emulsions were prepared from distilled water, Tween^®®^ 40 and AF using a combination of heating (80 °C, 10 min, stirring) and sonication (15 min) using VWR USC300T sonicator (VWR International, Radnor, PA, USA).

### 2.4. Humidity and Substrate Utilization Analysis

The obtained fermented bioproducts were collected and the humidity was measured. The humidity of substrates was measured by Moisture Analyzer Radwag 50/1. R (Radwag, Radom, Poland). Subsequently, the substrates were dried at 65 °C until a constant weight was achieved. The substrate utilization was calculated from the weight difference of the dry non-fermented substrate (control) and dry fermented bioproducts. Each sample was homogenized using a blender and stored in sterile Falcon tubes at laboratory temperature before the analysis was performed.

### 2.5. Estimation of Fungal Biomass in Fermented Bioproduct

To estimate the amount of fungal biomass in the fermented bioproducts, the method based on the estimation of glucosamine (GlcN) content was used [30,31]. In the first step, alkali insoluble material (AIM) was prepared according to Zamani et al. [24]: 0.5 M NaOH solution (3 mL) was added to 100 mg of each sample of fermented BP and the mixtures were heated at 90 °C for 16 h. Subsequently, samples were centrifuged (5000 rpm, 10 min) and washed 5 times with distilled water. Supernatants were removed and obtained AIMs were dried for 36 h at 75 °C. Further, AIM samples were hydrolyzed by adding 5 mL of 6M hydrochloric acid and incubating at 100 °C for 12 h. The analysis of glucosamine (GlcN) from the hydrolyzed AIM samples was performed spectrophotometrically according to Aidoo et al. [30] and GlcN content was calculated according to the standard calibration curve. Each analysis was performed in three independent technical replicates.

### 2.6. Microspopic Observation of Fungal Mycelia during SSF

The snapshots of fermented bioproducts were obtained by Dino-Lite Pro USB microscope. Fungal mycelia of *M. alpina* was cut out from the SSF matrix, suspended in 500 µL of sterile distilled water and observed by a confocal microscope Leica DM750 (Leica microsystems Ltd., Wetzlar, Germany), equipped with Leica HI PLAN 100x/1.25 oil objective lens (Leica microsystems Ltd., Wetzlar, Germany). Snapshots of individual hyphae were carried out with a Leica DFC290HD digital camera (Leica microsystems Ltd., Wetzlar, Germany).

### 2.7. Analysis of Fatty Acid Profile Andf Content in Fermented Bioproduct

Fatty acids (FA) from the SSF matrixes were converted into their methyl esters (FAMEs) by a modified method of Čertík and Shimizu [32]: 20 mg of dry homogenized bioproducts were mixed with 1 mL of dichloromethane containing 0.1 mg of heptadecanoic acid as an internal standard and 2 mL of anhydrous methanolic HCl solution. Samples were incubated at 50 °C for 3 h. After cooling down, 1 mL of distilled water was added and FAMEs were extracted with 1 mL of hexane. FAMEs were subsequently analyzed by gas chromatography according to the method described by Gajdoš et al. [33]. The identification of the FAMEs peaks was performed by comparison with authentic standards of C4-C24 FAME mixtures (Sigma Aldrich, USA). Quantitative evaluation of individual and total fatty acids was performed using an internal standard of heptadecanoic acid (C17:0, Sigma-Aldrich, Darmstadt, Germany) and calculated by ChemStation B 01 03 (Agilent Technologies, Santa Clara, CA, USA). Each analysis was performed in three independent technical replicates.

### 2.8. Lipid Isolation and Analysis of Lipid Classes by TLC

The lipids for the analysis of lipid classes were extracted with chloroform/methanol (2:1, *v*/*v*) according to original Folch et al. method [34] modified by Klempová et al. [15]. The lipid extracts were loaded on TLC silica plates 60 (Merck, Germany) using CAMAG TLC Sampler 4 (CAMAG, Muttenz, Switzerland) and TLC analysis was performed according to the method described by Gajdoš et al. [33]. Separated lipid fractions were analyzed densitometrically using CAMAG TLC Scanner 4 (CAMAG, Muttenz, Switzerland) and quantified using WinCATS software (CAMAG, Muttenz, Switzerland). Each analysis was performed in three independent technical replicates.

### 2.9. ATR-FTIR Spectroscopy Analysis

Fourier transform infrared (FTIR) spectroscopy was done employing an attenuated total reflectance (ATR) accessory for profiling the total biochemical composition of the obtained fermented bioproducts. ATR-FTIR measurements were performed using a Vertex 70 FTIR spectrometer (Bruker Optic, Billerica, MA, USA) with a single-reflection attenuated total reflectance (SR-ATR) accessory. The ATR-FTIR spectra were recorded with 32 scans using a horizontal SR-ATR diamond prism with 45° angle of incidence on a Specac (Slough, UK) High Temperature Golden Gate ATR Mk II. Of each homogenized dried fermented sample and of each non-fermented substrate sample, 10 mg of sample mass was prepared by homogenization with 2.8 mm stainless steel grinding balls (OPS Diagnostics, USA) using tissue homogenizer (5800 rpm, cycle 2 × 15 s with a 30 s pause) Percellys Evolution (Bertin Instruments, France). The samples were subsequently transferred to the surface of the ATR crystal. All ATR-FTIR measurements were performed in five technical replicates, resulting in 360 spectra in total. Spectra were recorded in a region between 7000 and 600 cm^−1^ with a resolution of 4 cm^−1^. Each spectrum was recorded as the ratio of the sample spectrum to the spectrum of the empty ATR plate. The recording of spectra was performed using the OPUS 7.5 software (Bruker Optic, Billerica, MA, USA).

### 2.10. Data Analysis

The fatty acid GC-FID and GlcN data were analysed by ANOVA using Microsoft Excel (Microsoft Office 365 software pack) equipped with a data analysis tool. Post-hoc testing was performed for the ANOVA results using Tukey’s HSD test in programming language R and in Python v. 3.7 using StatsModels libraries.

FTIR-ATR spectra were obtained for the region of 4000–600 cm^−1^, selected as the spectral region containing bands distinctive for lipids, proteins and polysaccharides. The analysis of FTIR-ATR spectra was performed by first applying pre-processing and then principle component analysis (PCA). The pre-processing was performed by transforming to second-derivative spectra using the Savitzky−Golay algorithm with a polynomial of degree 2 and a window size of 11. The second-derivative spectra were pre-processed by extended multiplicative signal correction (EMSC) [35,36]. PCA was performed for three spectral regions, lipid (3050–2800 cm^−1^ combined with 1800–1700 cm^−1^), protein (1700–1500 cm^−1^) and polysaccharide (1200–700 cm^−1^), using 11 principle components. The following software packages were used for data analysis: Unscrambler X version 10.5.1 (CAMO Analytics, Norway), Orange data mining toolbox version 3.24 (University of Ljubljana, Slovenia) [37].

## 3. Results

The present study is focused on utilization of AF by-products by the process of SSF employing fungus *M. alpina*. We evaluated two single-component control substrates, eight two-component control substrates containing Tween^®®^ 40 and eight three-component animal fat-based substrate mixtures (Table 1). Since emulsifier Tween^®®^ 40 may have impacted the fungal metabolism, the effect of Tween^®®^ 40 itself was also investigated.

### 3.1. Fungal Growth and Aubstrate Utilization during SSF

It was observed that the presence of Tween 40 at different concentrations in cereal-based substrates had no significant impact on the substrate utilization, substrate humidity, pH, biomass growth and FA content (*p*-value > 0.4; F parameter > F_crit_) (Table 2) and productivity was comparable with the control substrates based on cereal matrixes only.

It was also found that the utilization of control substrates and AF-based substrates in SSF varied depending on the type of cereal matrix used and the amount of AF added (Table 3). Generally, higher substrate utilization and fungal biomass growth was observed for the single-component and two-component substrates. The highest substrate utilization (25.8%) was reached for the substrates containing cornmeal (CM) and 1% of Tween (Table 3) and the highest fungal growth (289.3 mg/g of bioproducts) was obtained for the substrate containing CM with 3% of Tween. The utilization of CM single- and two-component control substrates was almost twice higher than for the respective wheat bran (WB) substrates (Table 3). Similarly, the fungal growth was 1.5-times higher using CM comparing to WB.

### 3.2. Microscopic Observation of SSF Process

**Figure 1:**
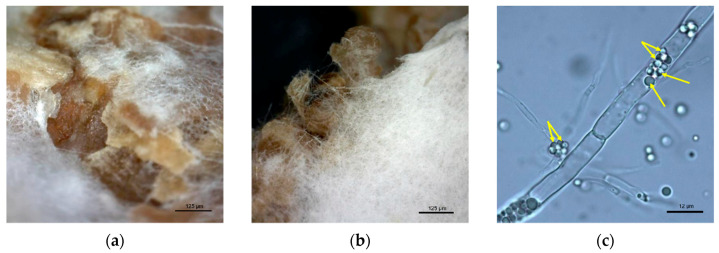
Microscopic images of *M. alpina* hyphae covering wheat bran (**a**), wheat bran with animal fat during solid-state fermentation (**b**) and lipid droplets accumulated inside *M. alpina* hyphae (**c**).

Fungal growth during SSF was also observed microscopically (Figure 1a,b). Similar morphology characteristics of *M. alpina* grown on control single- and two-component substrates and AF supplemented substrates were found. *M. alpina* was able to completely cover the substrate surface and its hyphae also sufficiently penetrated inside the substrate. Moreover, the presence of lipid structures formed in individual fungal hyphae was detected (Figure 1c).

### 3.3. The Impact of AF Supplementation on the Humidity and pH of the Fermented Bioproducts

It was found that increasing the amount of AF in substrate mixtures reduced the humidity of the fermented bioproducts (Table 3). Fermented bioproducts obtained from the SSF of control substrates had pH 5.3 (CM) and pH 5.5 (WB), while fermented bioproducts obtained from SSF of AF-based substrates showed lower pH values. The increase in AF amounts in fermentation substrates strongly affected the pH values of fermented bioproducts and led to the gradual reduction of pH in the SSF system (Table 3). The lowest pH (4.3) was observed for both cereal-based fermented bioproducts with a 30% (*w*/*w*) AF supplementation.

### 3.4. Lipid Profile and Accumulation

We observed that for the production of fatty acid-rich fungal biomss the CM is a more suitable substrate compared to WB (Table 3). Although the total fatty acid content in both substrates was similar, the total fatty acids content in bioproducts derived from SSF of single and two-component control substrates based on CM was almost four times higher in contrast to WB-based bioproducts. The total fatty acid content in fermented WB-based bioproducts (single- and two- component) was always lower after fermentation (Table 4). In all three-component bioproducts, the accumulation of fatty acids was strongly affected by the presence of AF, since the added AF was homogenized with other substrate components (Table 4). In addition, it was observed that the total fatty acid content of fermented bioproducts still varied depending on the cereal matrix used. When CM was used as a matrix, the total fatty acid content in fermented bioproducts was elevated compared to the non-fermented substrate. When WB was used as a matrix, the total fatty acid content dropped in fermented bioproducts comparing to the non-fermented substrate. Nevertheless, these results suggest that fungal strain *M. alpina* CCF2861 is definitively able to utilize exogenous fatty acids of AF by-product added to the substrate not only for its own fatty acids biosynthesis, but for hyphae proliferation and growth as well.

### 3.5. Arachidonic Acid Yield

**Figure 2:**
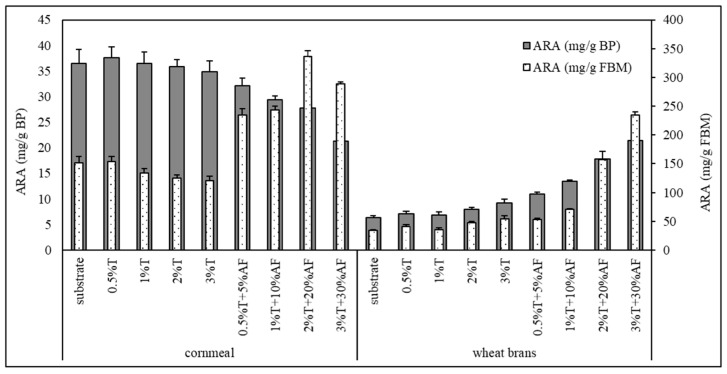
Content of arachidonic fatty acid (ARA) in fermented bioproducts (BP) and in fungal biomass (FBM). T—Tween 40, AF—animal fat by-product.

Comparing the total fatty acid profile of the fermented bioproducts we made the following observations (Table 4): (i) the percentage of ARA of total fatty acid content in bioproducts derived from the CM single- and two-component substrates was higher than for WB-based bioproducts; (ii) the total fatty acid profile of bioproducts derived from AF containing substrates was strongly affected by the fatty acid composition of the added AF—higher content of palmitic and stearic acid provided lower content of linoleic acid; (iii) the addition of the AF by-product to the SSF substrates resulted in the decrease of ARA content in the fermented BPs, where the degree of decrease was higher in CM-based bioproducts than in WB-based bioproducts. The levels of ARA in total fatty acids of bioproducts derived from CM-based substrates dropped from 26.5% (average value of single- and two-component substrates) to 8.1%, depending on the AF addition. On the other hand, the percentage of ARA of total fatty acids derived from WB-based bioproducts varied from 21.4% (average of single- and two-component substrates) to 12.6%, depending on the AF addition (Table 3). Comparing of bioproducts derived from single- and two-components substrates, it is clear that CM-based substrates were much more suitable for ARA biosynthesis than WB-based substrates (Figure 2). The average yield of ARA achieved using CM-based substrates was 36.2 mg ARA/g of BP (136.9 mg of ARA/g of fungal biomass), that is almost five times higher than the content of ARA in WB-based bioproducts, where the average yield from single- and two-component bioproducts was 7.5 mg ARA/g of bioproducts (42.2 mg ARA/g of fungal biomass). The addition of AF affected the ARA yield significantly (Figure 2). It caused a gradual cessation of ARA yield in CM-based bioproduct from 32.1 mg of ARA/g of bioproduct (addition of 5% (*w*/*w*) of AF) down to 21.2 mg ARA/g BP (addition of 30% (*w*/*w*) of AF). However, the content of ARA in bioproducts from the three-component CM-based substrates was at least double comparing to single- and two-components substrates. On the other hand, the addition of AF increased the yield of ARA in WB-based bioproducts up to 21.4 mg ARA/g of bioproduct (234.4 mg ARA/g of fungal biomass) in comparison to the fermented bioproducts from single-component substrates (7.1 mg ARA/g of bioproduct; 36.2 mg ARA/g of fungal biomass (Figure 2)).

### 3.6. Biochemical Profile of the Fermented Bioproducts Obtained by FTIR-ATR Spectroscopy

**Figure 3:**
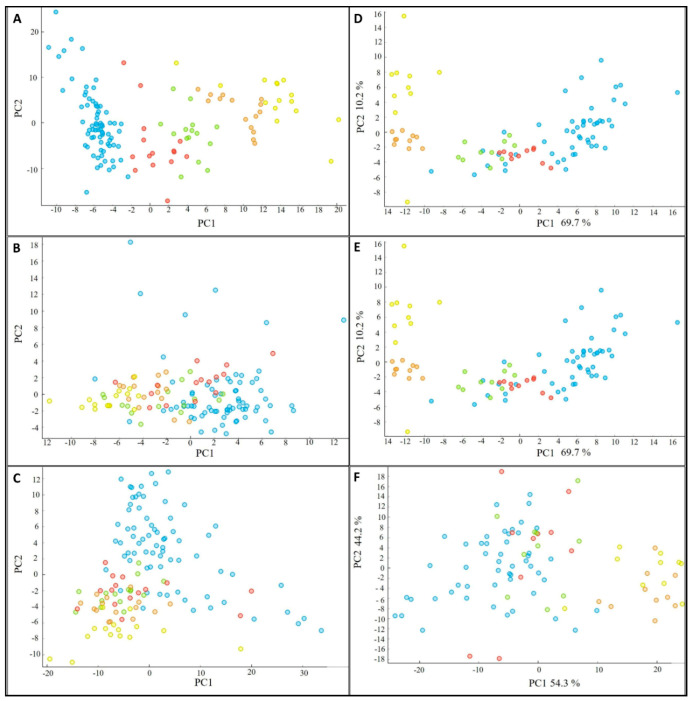
PCA scatter plots of FTIR-ATR spectra of fermented bioproducts obtained from the SSF of cornmeal-based and wheat bran-based substrates supplemented with animal fat at concentrations (blue) 0%, (red) 5%, (green) 10%, (orange) 20% and (yellow) 30%. (**A**)—lipid region of cornmeal-based fermented substrates, (**B**)—protein region of cornmeal-based fermented substrates, (**C**)—polysaccharide region of cornmeal-based fermented substrates, (**D**)—lipid region of wheat bran-based fermented substrates, (**E**)—protein region of wheat bran-based fermented substrates, (**F**)—polysaccharide region of wheat bran-based fermented substrates.

FTIR-ATR spectroscopy allows rapid and non-destructive analysis of the total biochemical composition of different biological materials; in our case—bioproducts obtained by SSF of *M. alpina*. Principal component analysis (PCA) of obtained FTIR-ATR spectra was used to study variations in the biochemical profile of the fermented bioproducts. The PCA scatter plot of the lipid-related spectral regions (3050–2800 cm^−1^ and 1800–1700 cm^−1^) shows significant chemical differences in the lipid profile for the individual fermented bioproducts obtained in SSF of substrates with various addition of AF (Figure 3). It was observed that the FTIR lipid profile of bioproducts obtained from the WB-based substrates was quite similar to the lipid profile of bioproducts derived from the substrates with 5% and 10% (*w*/*w*) of AF, while the lipid profile of bioproducts from the substrates with 20% and 30% (*w*/*w*) of AF showed a very different lipid profile (Figure 3). On the other hand, in case of CM-based bioproduct, PCA score plot (Figure 3) shows large differences between all tested conditions. The protein and polysaccharide profile of the fermented bioproduct from the SSF of AF supplemented substrates was similar, while it was different from the control substrates (Figure 3). The complete biochemical profile of bioproducts from the SSF of the control CM substrates was more similar to the CM substrates supplemented with 5% and 10% (*w*/*w*) of AF than in the case of WB substrates (Figure 3).

## 4. Discussion

Oleaginous fungus *Mortierella alpina* is a well-known and thoroughly described species with a high capacity for lipid accumulation and a good ability to produce industrially relevant essential PUFAs [19,20,21]. In order to verify growth ability and utilization of AF by-products by *M. alpina* in SSF, two types of cereal matrixes (wheat bran and cornmeal) were used in this study. Cereal-based matrixes are easily accessible and well-known as a good matrix for SSF substrates. Due to their chemical composition, such as presence of easily accessible carbon, organic nitrogen and other macro- and micro-nutrients, cereal-based matrixes provide a useful source for good fungal proliferation, hyphae penetration and stable lipid accumulation in fungal cells [10,15]. In order to improve the conversion of cereal-based carbon into PUFAs enriched bioproducts, supplementation with exogenous oils containing precursors of PUFAs is advised. Thus, the addition of vegetable oils, such as sunflower, rapeseed, corn, soybean or linseed oil containing individual fatty acid precursors of PUFAs, led to a rapid increase of PUFAs yield in the final fermented bioproducts obtained by fungal SSF [10]. It was also reported that *M. alpina* is able to incorporate and transform exogenous fatty acids [22]. In this study, an addition of animal fat by-product as a source of PUFA precursors (such as palmitic acid, stearic acid or oleic acid) to the cereal-based matrix was evaluated for the production of ARA-enriched fermented bioproducts in an SSF by *M. alpina* CCF2861.

Due to the fact that cereal-based matrixes have a hydrophilic nature while animal fat is hydrophobic, there is a need to perform pretreatment of the fat materials before mixing it with cereal matrixes for obtaining homogenous substrates for SSF. The pretreatment of the AF by-product was performed by preparing homogenous fat-water-Tween^®®^ 40 emulsions, where emulsifier Tween^®®^ 40 forms a film around the dispersed lipid droplets and thereby reduces interfacial tension [29].

While the total carbon content in both substrates is very similar (37.4% for CM and 37.9% for WB), the difference is in the profile of the dominant carbon source. The main carbon source in cornmeal is starch accounting for up to 85% (*w*/*w*) of the total carbon, whereas wheat bran contains only up to 24.5% of starch and up to 50% of cellulose and hemicellulose [38,39]. *M. alpina* is known to effectively use starch as a carbon source, however the cellulose and hemicellulose polymers are not suitable source of carbon for this strain [40]. This might be a reason for better fungal growth of *M. alpina* on CM in contrast to WB. Analysis of protein content using the automatic Kjeldahl method for nitrogen content showed that WB contained higher amount of proteins (17.4%) than CM (6.5%), indicating lower C/N ratio in WB than in CM. However, the amount of proteins was sufficient for fungal growth and proliferation in SSF process for both substrates.

Nevertheless, as mentioned above, fungal growth in the presence of AF was undoubtedly affected mostly by the carbon structure (CM—starch, WB—cellulose and hemicellulose polymers), significant changes in pH values were also noticed. Subsequent TLC analyses of lipid classes of extracted total lipid clearly indicated that AF contains relatively higher levels of free fatty acids (Table 1), which was probably the reason for lower pH of the fermented bioproducts after cultivation with AF addition.

Fatty acid analysis of all fermented bioproducts obtained in this study confirmed the previously described good ability of *M. alpina* to produce ARA (Figure 2, Table 3) [4,20]. High ARA yield in the fermented bioproducts obtained after SSF of AF supplemented substrates proves a high potential of applying *M. alpina* for the biotransformation of AF by-products into high-value ARA-enriched fermented bioproducts.

FTIR-ATR spectroscopy performed on all obtained fermented samples has proved possible high potential of this method for analysis of different types of materials. This method requires low sample amounts and does not involve extraction of any chemical components [26]. The infrared (IR) spectrum contains information about chemical bonds characteristic to all major biomolecules of the measured sample and different spectral regions representing information about lipids, proteins, phosphate containing molecules and polysaccharides. When analyzing cellular lipid profile based on FTIR spectra, the most important lipid associated peaks, used for the analysis, are: (i) peaks related to -CH_3_ and -CH_2_ stretching at 2947 cm^−1^, 2925 cm^−1^, 2855 cm^−1^, 1465 cm^−1^ and 1377 cm^−1^, indicating mainly the chain length of the carbon skeleton in lipid molecules; (ii) the peak related to the ester bond stretching at 1745 cm^−1^, indicating the total lipid content in the cell; (iii) the peak related to the carboxyl bond vibrations in free fatty acids at 1710 cm^−1^, and (iv) the peak related to =C-H stretching at 3010 cm^−1^, indicating the unsaturation level of cellular lipids. Proteins are observed in the spectral region 1700–1500 cm^−1^ with the main peaks for amide I (1650 cm^−1^) and amide II (1540 cm^−1^) bonds and polysaccharides are observed in the region 1200–900 cm^−1^. PCA of FTIR-ATR spectra proved distinct variations between individual samples in accordance with different composition of analyzed fermented samples. PCA score plots show large difference between all tested conditions, which could be caused by different composition of cereal substrate itself, as mentioned above. Higher similarity of complete biochemical profile in CM control samples with the CM substrates supplemented with 5% and 10% (*w*/*w*) of AF than in the case of WB substrates can be explained by the higher homogeneity of fungal growth and various cereal-based substrate utilization for the WB-based and CM-based substrates.

This is the first study on effective utilization of AF by-product into functional high-valued fermented bioproducts by SSF and obtained results represent broad fundamentals for subsequent research. Optimization of such fermentation process and extension for application of different fungal strains can lead to increased yields of desired metabolites or formatting a various high-value fungal specific product. Moreover, successful application of ATR-FTIR spectroscopy for rapid analysis of SSF bioproducts has been described, which can possibly extend industrial potential of the whole solid-state fermentation technology.

## Figures and Tables

**Table 1:** Fatty acid (FA) composition and percentage of individual lipid components of animal fat (AF) by-products used for the solid-state fermentation by *Mortierella alpina.*

FA	[%]
C14:0	2.23
C16:0	25.66
C16:1 n-7	2.18
C18:0	21.33
C18:1 n-9	38.34
C18:1 n-7	1.94
C18:2 n-6	5.47
C18:3 n-3	0.81
C20:0	0.23
Other fatty acids	1.81
**Lipid Structure**	
Polar lipids	0.14
Monoacylglycerols	0.28
Diacylglycerols	2.08
Sterol structures	9.38
Free fatty acids	7.20
Triacylglycerols	66.54
Esterified sterols	11.23
Other lipid structures	3.15

**Table 2:** Amount of added Tween^®®^ 40 and animal fat to cereal matrixes used for the solid-state fermentation by *Mortierella alpina.*

Cereal Matrix	Tween^®®^ 40 [%(*w*/*w*)]	Animal Fat [%(*w*/*w*)]
cornmeal	0	0 ^1^
	0.5	0 ^2^
	1	0 ^2^
	2	0 ^2^
	3	0 ^2^
	0.5	5 ^3^
	1	10 ^3^
	2	20 ^3^
	3	30 ^3^
wheat bran	0	0 ^1^
	0.5	0 ^2^
	1	0 ^2^
	2	0 ^2^
	3	0 ^2^
	0.5	5 ^3^
	1	10 ^3^
	2	20 ^3^
	3	30 ^3^

^1^ one-component samples (distilled water); ^2^ two-component samples (distilled water + Tween^®®^ 40); ^3^ three-component samples (distilled water + Tween^®®^ 40 + AF).

**Table 3:** Substrate utilization, substrate humidity, pH and fungal biomass (FBM) content in fermented bioproducts obtained from SSF of animal fat (AF) by-product by *Mortierella alpina*.

	Substrate Utilization [%]	Substrate Humidity [%]	pH	FBM [mg/g BP]
cornmeal (CM)	22.3 ± 0.9	60.0 ± 0.5	5.4 ± 0.1	241.3 ± 10.5
CM + 0.5% Tween 40	23.1 ± 0.5	58.1 ± 0.6	5.5 ± 0.0	244.0 ± 26.8
CM + 1% Tween 40	25.8 ± 0.5	60.1 ± 0.7	5.3 ± 0.1	272.8 ± 13.5
CM + 2% Tween 40	24.3 ± 0.6	59.0 ± 1.5	5.3 ± 0.3	286.2 ± 6.1
CM + 3% Tween 40	23.7 ± 1.0	58.6 ± 0.8	5.2 ± 0.1	289.3 ± 13.8
CM + 0.5% Tween 40 + 5% AF	21.1 ± 1.4	53.2 ± 0.6	5.2 ± 0.0	137.1 ± 15.6
CM + 1% Tween 40 + 10% AF	17.6 ± 0.4	48.8 ± 1.3	4.6 ± 0.1	120.7 ± 10.3
CM + 2% Tween 40 + 20% AF	13.1 ± 0.4	40.0 ± 1.1	4.5 ± 0.2	82.7 ± 14.8
CM + 3% Tween 40 + 30% AF	10.7 ± 0.4	36.5 ± 1.5	4.3 ± 0.1	73.7 ± 6.0
wheat bran (WB)	13.2 ± 0.5	58.7 ± 1.9	5.5 ± 0.1	188.8 ± 1.4
WB + 0.5% Tween 40	12.3 ± 0.6	58.9 ± 0.1	5.1 ± 0.5	174.2 ± 10.4
WB + 1% Tween 40	12.5 ± 0.4	56.8 ± 1.9	5.3 ± 0.2	193.6 ± 5.2
WB + 2% Tween 40	12.6 ± 0.7	58.4 ± 0.2	5.2 ± 0.2	168.1 ± 5.9
WB + 3% Tween 40	13.6 ± 0.6	54.2 ± 4.5	5.4 ± 0.3	166.0 ± 1.1
WB + 0.5% Tween 40 + 5% AF	10.4 ± 0.4	54.4 ± 1.0	5.1 ± 0.2	205.0 ± 1.1
WB + 1% Tween 40 + 10% AF	9.6 ± 0.2	48.7 ± 1.6	4.8 ± 0.1	189.9 ± 4.8
WB + 2% Tween 40 + 20% AF	9.3 ± 0.7	44.2 ± 2.2	4.5 ± 0.1	122.7 ± 5.1
WB + 3% Tween 40 + 30% AF	8.8 ± 0.5	39.1 ± 0.7	4.3 ± 0.0	92.2 ± 5.2

**Table 4:** Total fatty acid (TFA) content and fatty acid composition of non-fermented (nf) and fermented (f) cereal substrates (cornmeal—CM, wheat bran—WB) with the addition of different amounts of Tween 40 and animal fat (AF). The results are average of three independent biological replicates with α < 5%.

	TFA	Fatty Acids (%)											
	(%/BP)	C14:0	C16:0	C16:1, n-7	C18:0	C18:1, n-9	C18:1, n-7	C18:2, n-6	C18:3, n-6	C18:3, n-3	C20:3, n-6	C20:4, n-6	Others
cornmeal													
CM nf	3.2	0.6	12.7	nd	2.5	27.3	0.7	54.7	nd	1.5	nd	nd	nd
CM f	13.8	0.8	14.5	0.1	5.6	13.7	0.7	23.8	2.3	0.5	2.5	26.4	9.0
+0.5%Tween 40 nf	3.7	0.7	14.3	nd	2.4	26.2	0.7	54.1	nd	1.5	nd	nd	nd
+0.5%Tween 40 f	13.7	0.6	15.6	0.1	5.8	13.2	1.0	23.5	2.4	0.3	2.5	27.5	7.6
+1%Tween 40 nf	3.4	0.8	16.6	nd	2.6	25.4	0.7	52.4	nd	1.6	nd	nd	nd
+1%Tween 40 f	13.4	0.8	16.0	0.1	5.7	12.8	0.6	22.6	2.3	0.4	2.6	27.1	8.9
+2%Tween 40 nf	4.3	1.0	20.3	nd	2.6	23.7	0.6	50.2	nd	1.5	nd	nd	nd
+2%Tween 40 f	13.8	0.8	17.3	0.1	5.8	13.0	0.6	22.7	2.3	0.5	2.5	26.1	8.4
+3%Tween 40 nf	4.7	0.6	23.9	nd	2.8	22.3	0.6	48.3	nd	1.3	nd	nd	nd
+3%Tween 40 f	13.7	0.8	18.2	0.1	5.7	12.7	0.6	23.0	2.3	0.5	2.5	25.4	8.2
+0.5%T + 5%AF nf	7.1	0.7	18.0	0.8	7.8	30.9	1.1	39.4	nd	1.3	nd	nd	nd
+0.5%T + 5%AF f	14.2	1.0	17.1	0.6	8.8	18.0	1.2	18.6	2.1	0.6	1.8	22.6	7.5
+1%T + 10%AF nf	11.3	1.2	22.2	1.7	12.0	33.4	1.5	26.9	nd	1.2	nd	nd	nd
+1%T + 10%AF f	16.1	1.1	18.8	1.0	11.3	21.1	1.5	16.4	2.0	0.6	1.4	18.3	6.4
+2%T + 20%AF nf	18.1	1.7	24.7	2.0	15.0	36.4	1.7	17.5	nd	1.1	nd	nd	nd
+2%T + 20%AF f	19.0	1.4	21.0	1.3	13.5	24.8	1.7	12.9	1.6	0.7	1.1	14.6	5.3
+3%T + 30%AF nf	24.3	1.9	25.8	2.1	16.6	37.4	1.8	13.3	nd	1.0	nd	nd	nd
+3%T + 30%AF f	24.7	1.6	22.4	1.6	14.7	30.4	1.7	12.7	1.2	0.8	0.7	8.6	3.6
**wheat bran**													
WB nf	3.5	1.7	17.1	nd	1.0	17.3	1.5	56.1	nd	4.5	nd	nd	0.7
WB f	3.1	0.2	12.6	0.1	1.6	15.3	1.1	38.6	2.2	2.3	0.6	20.4	5.2
+0.5%Tween 40 nf	3.6	nd	20.7	nd	1.1	16.6	1.5	54.7	nd	4.4	nd	nd	0.9
+0.5%Tween 40 f	3.4	0.1	13.9	nd	1.5	15.9	1.1	38.2	1.8	2.6	0.3	20.5	4.1
+1%Tween 40 nf	3.8	nd	21.8	0.2	1.2	16.7	1.4	53.4	nd	4.2	nd	nd	1.2
+1%Tween 40 f	3.3	0.3	15.7	nd	1.7	14.4	1.1	36.8	2.3	2.2	0.6	20.6	4.3
+2%Tween 40 nf	3.8	nd	26.7	nd	1.5	15.8	1.4	50.1	nd	3.9	nd	nd	0.7
+2%Tween 40 f	3.6	0.3	15.5	nd	2.1	13.8	1.1	34.9	2.4	2.1	0.2	22.3	5.5
+3%Tween 40 nf	4.6	0.2	33.6	0.2	1.8	13.7	1.2	44.9	nd	3.5	nd	nd	1.0
+3%Tween 40 f	4.0	0.3	18.8	0.2	2.3	13.0	1.0	31.5	2.1	2.0	0.7	23.1	5.1
+0.5%T + 5%AF nf	6.6	1.0	22.1	1.0	9.5	26.5	1.6	34.6	nd	2.9	nd	nd	0.8
+0.5%T + 5%AF f	5.6	0.5	17.2	0.7	7.1	18.2	1.9	21.8	3.5	1.2	0.8	19.6	7.4
+1%T + 10%AF nf	9.9	1.2	23.7	1.2	10.6	27.2	1.7	30.7	nd	2.6	nd	nd	1.1
+1%T + 10%AF f	7.6	0.8	17.7	1.0	8.6	22.9	1.91	17.7	3.6	1.2	0.8	18.1	5.9
+2%T + 20%AF nf	14.4	1.8	25.8	1.7	12.8	31.1	1.8	22.2	nd	2.0	Nd	nd	1.1
+2%T + 20%AF f	11.4	1.1	19.6	1.3	11.6	24.2	2.0	13.8	4.0	1.0	0.9	15.6	5.9
+3%T + 30%AF nf	23.4	1.3	26.1	1.6	16.0	33.3	1.8	17.1	nd	1.6	nd	nd	0.9
+3%T + 30%AF f	17.0	1.0	19.8	1.3	13.3	25.9	2.0	13.6	3.7	1.0	0.8	12.6	5.1
